# Psychological Determinants of Healthy Food Purchase Intention: An Integrative Model Based on Health Consciousness

**DOI:** 10.3390/nu17071140

**Published:** 2025-03-26

**Authors:** Manuel Escobar-Farfán, Elizabeth Emperatriz García-Salirrosas, Mauricio Guerra-Velásquez, Iván Veas-González, Ledy Gómez-Bayona, Rodrigo Gallardo-Canales

**Affiliations:** 1 Department of Administration, Faculty of Administration and Economics, University of Santiago of Chile (USACH), Santiago 9170020, Chile; manuel.escobar@usach.cl (M.E.-F.); mauricio.guerra@usach.cl (M.G.-V.); 2 Faculty of Management Science, Universidad Autónoma del Perú, Lima 15842, Peru; 3 Departamento de Administración, Facultad de Economía y Administración, Universidad Católica del Norte, Antofagasta 1270709, Chile; iveas@ucn.cl; 4 Instituto Tecnológico Metropolitano, Facultad Ciencias Económicas Administrativas, Medellin 050034, Colombia; ledygomez@itm.edu.co; 5 Departamento de Tecnologías de Gestión, Facultad Tecnológica, Universidad de Santiago de Chile (USACH), Santiago de Chile 9170125, Chile; rodrigo.gallardo@usach.cl

**Keywords:** health consciousness, purchase intention, perceived behavioral control, self-identity, moral norms, healthy food, theory of planned behavior

## Abstract

**Background/Objectives**: Health consciousness has emerged as a key driver of healthy food purchase decisions in the post-pandemic era. Despite growing interest in health-oriented products, the psychological mechanisms through which health consciousness influences purchase intentions remain understudied. This research examined how health consciousness impacts healthy food purchase intentions through multiple psychological pathways, integrating the Theory of Planned Behavior with additional constructs. **Methods**: Data were collected through an online survey of 573 Peruvian consumers. Healthy foods were operationalized based on their nutritional quality, including a high nutrient content, low saturated fats and added sugars levels, and minimal processing. Structural equation modeling was employed to test the hypothesized relationships between health consciousness, attitudes, perceived behavioral control, self-identity, moral norms, and purchase intention. **Results**: Health consciousness demonstrated significant direct effects on all psychological mediators (attitudes: β = 0.643; perceived behavioral control: β = 0.593; self-identity: β = 0.638; moral norms: β = 0.613) and purchase intention (β = 0.163). However, only perceived behavioral control (β = 0.261) and self-identity (β = 0.107) significantly influenced the purchase intention, while the effects of attitudes and moral norms were non-significant. **Conclusions**: The findings challenge traditional assumptions about the primacy of attitudes in consumer decision making and highlight the importance of perceived behavioral control and self-identity in translating health consciousness into purchase intentions. Successfully promoting healthy food consumption requires strategies addressing both practical barriers and identity-related aspects of food choice, providing valuable insights for food marketers and public health initiatives.

## 1. Introduction

The post-pandemic era has ushered in significant changes in consumer behavior toward healthy food consumption, with heightened health consciousness emerging as a key driver of purchase decisions [1,2,3,4,5,6]. This transformation is particularly evident in the functional food market, where consumers increasingly seek products that provide basic nutrition and offer additional health benefits and immune system support [3,7,8]. Understanding the psychological mechanisms underlying these changing consumption patterns has become crucial for both researchers and practitioners in the food industry.

For this study, healthy foods are defined as those with high nutritional quality according to their nutrient composition and processing level. Specifically, foods are considered “healthy” based on: (1) a high content of essential nutrients (vitamins, minerals, protein, and/or fiber); (2) low levels of saturated fats, trans fats, added sugars, and sodium; and (3) minimal processing that preserves the nutritional integrity. This definition encompasses fresh or minimally processed fruits and vegetables, whole grains, legumes, nuts and seeds, lean proteins, and low-fat dairy products. The nutritional basis for this definition draws from established nutritional science linking these food characteristics to positive health outcomes [9]. For example, an adequate intake of fruits and vegetables provides essential vitamins (A, C, E, and K) and minerals (potassium and magnesium) that support immune function and reduce inflammation [10,11]. In the Peruvian context, this definition includes both globally recognized healthy foods and nutrient-dense indigenous products such as quinoa, kiwicha, and native fruits like camu camu, which are rich in protein, fiber, antioxidants, and essential minerals [12].

The purchase intention represents the cognitive disposition of an individual to engage in a specific buying behavior. In the context of consumer behavior, it serves as a crucial prerequisite that drives consumers toward the acquisition of products and services [1,13,14,15,16,17,18]. In the specific context of healthy food, this concept refers to consumers’ predisposition to acquire products perceived as beneficial for their health [19,20,21]. Research on the purchase intention has been extensively conducted through various theoretical frameworks, particularly the Theory of Reasoned Action (TRA) [22] and its extension, the Theory of Planned Behavior (TPB) [23,24]. The TRA posits that attitudes and subjective norms are key antecedents of behavioral intentions, while the TPB extends this framework by incorporating perceived behavioral control as an additional predictor. These theoretical frameworks have been widely applied to understand consumer purchase intentions across various contexts, including healthy food consumption [25,26,27,28,29,30,31]. Empirical evidence has consistently demonstrated that the purchase intention significantly influences the actual purchasing behavior for healthy and organic foods [32,33,34]. Recent studies have further reinforced this relationship, showing that consumers’ perceptions of healthy foods directly impact their purchase intentions and subsequent buying behavior [34,35].

While the TPB provides a robust foundation for understanding purchase intentions, research has increasingly recognized the need to incorporate additional psychological constructs to better explain consumer behavior in the context of food choices [36,37,38]. Self-identity, defined as the extent to which performing a behavior is an essential component of an individual’s self-concept, has emerged as a significant predictor of intentions beyond the traditional TPB components [39,40,41]. For food-related behaviors specifically, consumers who identify themselves as health-conscious individuals show stronger intentions to purchase healthy foods, as this behavior aligns with their self-perception [42,43].

Similarly, moral norms—personal beliefs about right or wrong based on internalized values—have been identified as important determinants of food choice intentions [44,45,46]. In the context of healthy eating, these moral considerations often reflect consumers’ sense of responsibility toward their health and well-being [47,48]. Recent research by Askegaard et al. [49] and Arvola et al. [50] has demonstrated that moral norms can significantly enhance the predictive power of models explaining healthy food purchase intentions, particularly when there is a high level of health consciousness.

This study addresses critical research gaps by examining the complex psychological mechanisms through which health consciousness influences healthy food purchase intentions. While previous research has established the importance of health consciousness in consumer decision making, the interplay between various psychological factors and their collective influence on purchase intentions remain understudied [43,51,52,53,54]. By integrating attitude and perceived behavior control with additional psychological constructs such as moral norms and self-identity, this research provides a comprehensive framework for understanding healthy food purchase behavior determinants.

The timing of this investigation is particularly relevant as the food industry undergoes rapid transformation, with consumers becoming increasingly selective about healthier and more sustainable food choices [7,55,56,57]. The COVID-19 pandemic has accelerated these changes, leading to structural shifts in consumer preferences and market dynamics [2,55]. As emerging markets continue to grow and evolve, understanding the psychological drivers of healthy food consumption becomes increasingly important for both local and global food industry stakeholders [7,58]. Furthermore, this study has significant implications for practitioners in the food industry. As market research indicates a growing demand for healthier foods and beverages, the findings will help food marketers better understand consumer psychology and develop products that align with emerging preferences [57,59,60]. This understanding is crucial as consumers become more selective in food choices and increasingly prioritize personal and family health requirements [4,61,62]. Additionally, the research provides valuable insights for policymakers and health advocates working to promote healthier eating habits in the post-pandemic era.

Therefore, the primary objective of this study is to examine how health consciousness influences healthy food purchase intentions through multiple psychological mechanisms. Specifically, we aim to (1) assess the direct effect of health consciousness on purchase intentions; (2) examine how health consciousness influences key psychological constructs including attitudes, perceived behavioral control, self-identity, and moral norms; and (3) determine which psychological mechanisms are most effective in translating health consciousness into purchase intention. This research has significant international relevance as global health concerns reshape food consumption patterns across diverse markets. Our findings can inform marketing strategies and public health initiatives in emerging economies like Peru, where rapid urbanization and increasing health awareness drive shifts in dietary practices. Understanding these psychological mechanisms can help develop more effective interventions to promote healthy eating behaviors across different cultural contexts, contributing to global commercial sustainability and public health improvements. The findings will contribute to both the theoretical understanding and practical applications in several ways. First, it extends current theoretical frameworks in consumer behavior by examining health consciousness’s direct and indirect effects on purchase intentions. Second, the research addresses the identified gap in the literature regarding behavioral characteristics influencing healthy food consumption in the post-pandemic context [55]. Furthermore, this study has significant implications for food industry practitioners and policymakers working to promote healthier eating habits in the post-pandemic era.

## 2. Literature Review

Building upon these theoretical foundations, this study proposes an integrative model that examines the psychological determinants of healthy food purchase intentions. Health consciousness emerges as a fundamental construct influencing multiple aspects of consumer behavior related to healthy food consumption [51,60,63,64].

In the proposed model, Health Consciousness serves as an antecedent variable. Recent studies support this approach that Health Consciousness functions as a fundamental motivational factor that precedes and shapes psychological constructs like attitudes, perceived control, and self-identity [2,65]. We conceptualize Health Consciousness as a relatively stable consumer trait that forms the foundation for subsequent psychological responses rather than a variable that merely alters the strength of relationships between these constructs. This antecedent role of Health Consciousness aligns with the hierarchical model of consumer behavior, which positions health-related values and consciousness as primary drivers that influence subsequent psychological mechanisms [51].

**Figure 1:**
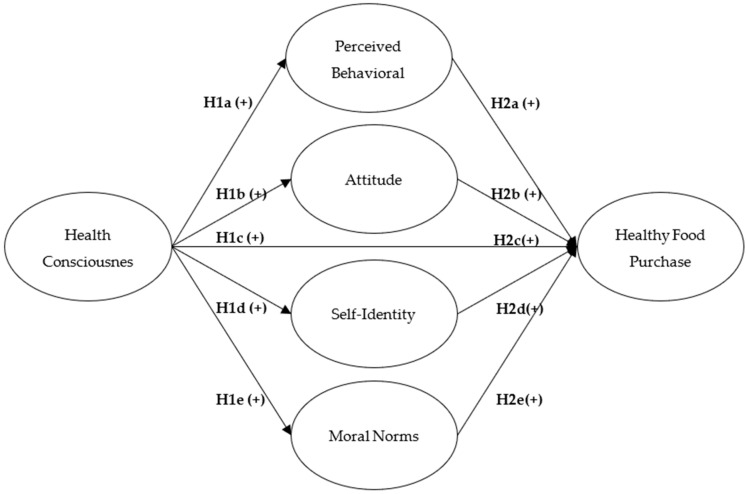
Proposed theoretical model of health consciousness influence on healthy food purchase intention.

This psychological factor directly affects the purchase intention and operates through various psychological and social mechanisms [2,43,66]. The Theory of Planned Behavior provides a robust framework for understanding how attitudes and perceived behavioral control influence these intentions [50,66,67], while additional constructs such as self-identity and moral norms complement and enrich this understanding [36,37,68]. These variables are not isolated, but form an integrated influence system that guides healthy food consumption decisions [31,39,49]. Based on this integrative approach, we propose a theoretical model in which health consciousness directly influences the purchase intention through multiple psychological mechanisms, including attitudes, perceived behavioral control, self-identity, and moral norms, as depicted in Figure 1.

### 2.1. Health Consciousness Positively Impacts Attitude Regarding Healthy Eating

Health consciousness and its influence on consumer attitudes have been extensively studied in food consumption research, particularly regarding healthy and sustainable food choices. Multiple studies across different geographical and cultural contexts have consistently demonstrated that health consciousness strongly predicts positive attitudes toward health-oriented food products [15,66,68]. Health consciousness emerges as one of the most significant factors shaping consumer attitudes toward more nutritious eating habits [69,70,71]. This finding is significant during the COVID-19 pandemic, where health consciousness has directly influenced consumer attitudes towards healthy food choices [3,6,15]. Recent empirical evidence has further reinforced this connection, demonstrating that heightened health consciousness influences attitudes and can lead to sustained healthy food preferences [35,51,63,72]. This is particularly relevant in health-oriented products, where consumer health consciousness significantly impacts attitude formation and subsequent food evaluations.

### 2.2. Health Consciousness Positively Impacts Perceived Behavioral Control Regarding Healthy Eating

Research has established that health consciousness evaluates an individual’s readiness to undertake health-related actions, particularly in food choice contexts [63,73]. Multiple studies across different contexts have consistently demonstrated that individuals exhibit greater perceived control over their food choices and dietary behaviors [74,75,76]. Health consciousness is key in enhancing individuals’ perceived ability to control their food choices. This finding is particularly significant as health-conscious consumers usually maintain their health and quality of life through healthy nutrition and physical fitness [35,72]. Studies show that consumers with higher health consciousness have greater perceived control over avoiding unhealthy food choices and opting for healthier alternatives [10,35,63]. Health consciousness influences perceived control and strengthens consumers’ confidence in their ability to make healthy food choices [10,35,42,77,78].

### 2.3. Health Consciousness Positively Impacts Healthy Food Purchase Intention

Regarding health consciousness, Wiedenroth and Otter [79] affirmed that this factor emphasizes how consumers gather health-related information and understand food benefits. Health-conscious individuals typically demonstrate greater involvement in health-promoting behaviors and show increased attention to nutrition information and product ingredients [35,73]. Research has consistently shown that health consciousness significantly influences consumer decision-making processes in food choices. Consequently, more health-conscious consumers demonstrate a greater intention to purchase organic or healthy products [79]. Qi and Ploeger [2] identified health consciousness as one of the most significant motivating factors for organic food consumption, particularly during health-critical periods such as the COVID-19 pandemic. Similarly, Dudziak and Kocira [65] found that health concerns are the primary drivers for purchasing organic or healthy foods, surpassing other factors such as environmental concerns or taste preferences. Furthermore, studies have demonstrated that health consciousness influences purchase intentions, information-seeking behavior, and product evaluation processes [62,80]. Health-conscious consumers spend more time researching product benefits, reading nutritional labels, and comparing healthy food options [68].

### 2.4. Health Consciousness Positively Impacts Self-Identity Regarding Healthy Eating

Health consciousness is crucial in shaping consumers’ self-identity as health-conscious individuals [42,43,51,81]. Individuals’ health consciousness leads to developing health-oriented self-identities. Health consciousness is a fundamental driver in developing consumers’ self-identity. This finding is particularly significant as consumers’ concern about maintaining personal or familial health can invoke their interest in seeking information and knowledge about healthy food [43,81], and developing self-identities aligned with healthy consumption patterns. Studies have shown that the perceived adverse effects of conventionally produced food can raise health concerns among individuals, leading them to develop identities as healthy food consumers [42]. Health consciousness influences self-identity formation and can lead to openness in changing consumption patterns [62,80]. This is particularly relevant in health-oriented products, where Kim [62] found the concept of self and identity significantly correlated with health consciousness. Similarly, Qasim et al. [42] found health consciousness to influence environmental self-identity and behavioral intentions significantly.

### 2.5. Health Consciousness Positively Impacts Moral Norms Regarding Healthy Eating

Research has established that consumers develop a complex system of ethical norms around food choices beyond nutritional properties [47,49,50]. Multiple studies have demonstrated that health consciousness significantly influences the development of moral norms regarding food choices, as consumers categorize foods not just by their health benefits, but through an ethical lens of “good” versus “bad” choices [49,82]. This finding is particularly significant as health-conscious consumers tend to translate their food decisions into behavioral rules where choosing “good” foods signifies being righteous, moral, and decent [47,48,50]. Studies demonstrate that individuals with higher health consciousness develop stronger moral norms about food choices, viewing their dietary decisions as moral obligations rather than mere preferences [46,49]. Health consciousness influences moral norm formation and can lead to the development of sustained ethical frameworks around food choices [10,49].

### 2.6. Attitude Positively Impacts Healthy Food Purchase Intention

Multiple studies across different geographical and cultural contexts have consistently demonstrated that consumer attitudes strongly predict purchase intention for health-oriented food products [38,83,84,85,86]. Attitude emerges as one of the most robust predictors of consumer intention to adopt healthier eating habits [17,70,87,88]. This finding is particularly significant in functional and organic food markets, where positive consumer perceptions have been shown to influence purchase likelihood directly [66,88]. Recent empirical evidence has further reinforced this connection, demonstrating that positive attitudes influence purchase intentions and can lead to actual purchasing behavior [41,89]. This is particularly relevant in environmentally and socially responsible products, where consumer attitudes have been shown to impact purchase decisions significantly [17,66,90,91].

### 2.7. Perceived Behavioral Control Positively Impacts Healthy Food Purchase Intention

Perceived behavioral control, a key construct in the Theory of Planned Behavior, refers to individuals’ perception of ease or difficulty in executing specific behaviors [92,93]. This concept has proven particularly relevant in healthy food purchase decisions, where consumers’ sense of control over their actions significantly influences their behavioral intentions [73,94]. The empirical evidence consistently demonstrates that individuals with higher perceived behavioral control exhibit stronger intentions to purchase healthy food products [54,95,96,97,98]. Furthermore, research has shown that this relationship extends to environmentally friendly healthy products, where more substantial perceived behavioral control leads to enhanced purchase intentions [83]. The influence of perceived behavioral control on purchase intention can be affected by various factors. For instance, clear product labeling enhances consumers’ perceived behavioral control [99], while difficulties in identifying organic food labels can negatively impact purchase intentions [100]. Additionally, the degree of autonomy in food selection and acquisition can influence this perception of control [54,101].

### 2.8. Self-Identity Positively Impacts Healthy Food Purchase Intention

Self-identity emerges as a significant predictor of healthy food purchase intention, maintaining its influence even when controlling for other psychological and social factors within the theory of the planned behavior framework [37,81,102]. The internalization of a health-conscious identity is a self-regulatory mechanism that guides food choices, playing a crucial role in forming and maintaining healthy eating behaviors [37,39]. For instance, environmental self-identity, which often overlaps with healthy consumer identity, directly influences purchase intentions and amplifies the effect of other consumption values on eating behavior [42,103,104]. Furthermore, studies have shown that self-identity’s influence extends beyond direct purchase intentions to shape broader consumer–brand relationships. Specifically, a strong health-oriented self-identity influences trust towards health food retailers and affects willingness to consume products from brands perceived as healthy [40,41]. This relationship underscores self-identity’s role as a fundamental internal motivating factor in shaping consumer preferences and choices [8,41].

### 2.9. Moral Norms Positively Impact Healthy Food Purchase Intention

Moral norms, grounded in individual ethical values and principles, have been identified as significant drivers of healthy food consumption decisions, operating independently of external social pressures [36,51,105,106]. This influence extends beyond simple purchasing decisions, as moral considerations often intersect with perceptions of sustainability and ethical production in healthy food choices. Research in sustainable and ethical food consumption has provided strong evidence for the role of moral norms in shaping purchase intentions. Studies examining fair-trade products have demonstrated that moral norms significantly influence purchase intentions for ethically produced foods [38,44,46]. This relationship is particularly evident in the organic food sector, where moral considerations are strong predictors of purchase intentions [46]. The integration of the theory of planned behavior with value-belief-norm theory has revealed moral norms as crucial determinants in natural food purchase intentions [45].

Therefore, Table 1 summarizes our research model’s nine hypotheses, their theoretical rationale, and key supporting references. The following sections develop each hypothesis in more detail.

## 3. Materials and Methods

### 3.1. Context and Measures

This research examines how health consciousness influences healthy food purchase intentions through multiple psychological mechanisms, including attitude, perceived behavioral control, self-identity, and moral norms. This study aims to empirically validate the relationships between these constructs and their role in shaping purchase intentions for healthy foods, contributing to a more comprehensive understanding of health-conscious consumer behavior.

The measurement instruments for this study were carefully selected based on established scales from the literature. Health consciousness was measured using seven items adapted from Teng and Lu [110] and Hansen et al. [51], which capture consumers’ awareness and concern for health-related matters. The remaining constructs were assessed using scales adapted from Yazdanpanah and Forouzani [67]. Precisely, attitude was measured using three items, perceived behavioral control with three items, self-identity with two items, and moral norms with two items. The purchase intention was evaluated using three items. All measurement items were assessed using a 5-point Likert-type scale, where “1” means “Totally disagree” and “5” means “Totally agree”, ensuring consistency across all variables.

Before the questionnaire was applied, a two-stage validation process was carried out. First, content validation was performed by consulting experts who reviewed the relevance and clarity of the items. This process confirmed the items’ suitability in the current context. Subsequently, a pre-test was conducted with a pilot sample to evaluate the comprehension and appropriateness of the questions. The pre-test was conducted with 30 Peruvian consumers from diverse ages and educational backgrounds to ensure the items were understood locally. The pre-test results confirmed the clarity and precision of all items, and no modifications to the initially proposed items were necessary.

### 3.2. Sample and Procedure

Data collection was conducted through a quantitative approach using a non-probabilistic convenience sampling method [111]. An online survey was developed and distributed through Google Forms, with participants providing informed consent before participation. The survey was conducted in Spanish, the official language of Peru, to ensure participants could fully comprehend all questions and provide accurate responses. The online survey method was chosen for its advantages, including broader reach, faster response rates, cost-effectiveness, and efficient data collection. The survey was distributed through social media platforms, leveraging these channels’ effectiveness in reaching potential participants. Data collection took place during the second half of 2023 in Peru. This study acknowledges potential biases inherent to the methodology. First, self-selection bias may be present, as individuals with a greater interest in healthy eating were likely more motivated to participate. Second, the online nature of the survey may have limited participation from lower socioeconomic strata with reduced internet access, particularly in rural areas. Third, distribution through social media platforms may have created a geographical concentration in urban areas, primarily Lima Metropolitan.

To minimize these potential biases, this study employed several data collection strategies. This study used diverse social media channels to reach different demographic segments, carefully screened responses for completeness and consistency, and compared early versus late respondents to check for potential response bias. Additionally, to ensure participants understood what constituted “healthy food”, the research provided the definition described in the Introduction at the beginning of the survey.

The final sample consisted of 573 respondents who reported being consumers of healthy food products. Table 2 presents the demographic characteristics of the participants. The sample was predominantly female (65.1%) and young, with 85.4% of participants under 25. Most respondents were pursuing or had completed a Bachelor’s degree (89.5%), and most were single (93.4%). Comparing these demographics to recent Peruvian census data, our sample represents a specific segment of the population—primarily urban, educated young adults—rather than being nationally representative. However, this demographic segment represents a relevant consumer group for healthy food markets in emerging economies like Peru, particularly given their growing purchasing power and increasing health consciousness.

While this demographic composition presents certain limitations in terms of the generalizability, it provides valuable insights into the health-conscious consumption patterns of young, educated consumers. To enhance the reproducibility of this research, the complete questionnaire, including all measurement items in both Spanish (as administered) and the English translation, is provided in Appendix A.

### 3.3. Statistical Analysis Process

The data analysis was conducted using a comprehensive two-software approach. IBM SPSS version 25 was employed for initial data processing, including demographic analysis and preliminary data screening to detect outliers and assess the multivariate normality [112]. The primary analysis was performed using Smart-PLS version 4.0, following the two-step approach recommended by Hair et al. [112] for evaluating measurement and structural models. The Partial Least Squares Structural Equation Modeling (PLS-SEM) method was selected for its capability to analyze complex multivariate relationships within the conceptual framework simultaneously [113,114,115]. This choice was particularly appropriate given PLS-SEM’s advantages in handling multiple relationships simultaneously and its ability to assess both direct and indirect effects in structural models [112,115,116].

For the measurement model assessment, this research evaluated psychometric properties, including convergent validity, which is considered appropriate when all indicators demonstrate loadings exceeding 0.7 [112]; discriminant validity, using both the Fornell–Larcker criterion and cross-loadings assessment [112,117]; and reliability measures via Cronbach’s alpha and composite reliability [112]. For adequate reliability, both the average variance extracted (AVE) and composite reliability (CR) values should surpass the threshold of 0.5 [118], with Cronbach’s alpha coefficient greater than 0.7, which is generally considered optimal. In factor-based methodologies, the resulting factor typically produces values comparable to composite reliability measurements [112].

For the structural model evaluation, the analysis assessed path coefficients (*p*-value and *t*-value) to determine statistical significance, a coefficient of determination (R^2^) to evaluate the model’s predictive relevance, and the root mean square residual (SRMR) to assess the overall model fit. A bootstrapping procedure with 5000 subsamples was implemented to ensure robust statistical inference, following established consumer behavior research practices for evaluating the statistical significance of both path coefficients and indirect effects [119]. Notably, PLS-SEM’s application in interdisciplinary research has been praised by behavioral researchers [120].

## 4. Results

### 4.1. Measurement Model Assessment

A detailed examination of the measurement model’s psychometric properties reveals strong reliability and validity across all constructs (Table 3). Attitude demonstrates exceptionally high reliability (α = 0.960, CR = 0.974) and convergent validity (AVE = 0.926), with factor loadings ranging from 0.956 to 0.966. Health consciousness also shows robust reliability (α = 0.950, CR = 0.959) and satisfactory convergent validity (AVE = 0.772), with consistent factor loadings (0.805–0.908) across its seven indicators. The remaining constructs—moral norms (α = 0.799, CR = 0.908, AVE = 0.831), perceived behavioral control (α = 0.849, CR = 0.909, AVE = 0.768), purchase intention (α = 0.904, CR = 0.940, AVE = 0.839), and self-identity (α = 0.868, CR = 0.938, AVE = 0.883)—all exceed the critical thresholds established in the literature.

A heterotrait–monotrait ratio (HTMT) analysis was conducted to assess the discriminant validity. According to established criteria, HTMT values should be below 0.85 to demonstrate discriminant validity between constructs. This study reveals that most HTMT ratios fall well below this threshold, supporting the discriminant validity of the constructs. The relationships between HC and other constructs show acceptable values, ranging from 0.532 (HC-PI) to 0.702 (HC-S). Similarly, PI demonstrates good discriminant validity with all constructs, ranging from 0.527 (ATT-PI) to 0.628 (PBC-PI). While two relationships approach the upper limit—MN with ATT (0.806) and MN with PBC (0.871)—they remain within acceptable ranges according to the more lenient criterion of 0.90. The relationship between MN and S (0.823) also falls within acceptable parameters. Overall, these results strongly support the discriminant validity of the measurement model (Table 4).

Additionally, Table 5 shows that the square root of each construct’s Average Variance Extracted (values on the diagonal) exceeds the correlations with the other constructs, thus fulfilling the Fornell–Larcker criterion [117]. For example, the square root value of the AVE for “Attitude” is 0.962, which is greater than all its correlations with the other constructs (ranging from 0.498 to 0.717). Similarly, health consciousness has a square root of AVE of 0.879, exceeding its correlations with other constructs (ranging from 0.496 to 0.643). This pattern holds for all constructs in the model, confirming the discriminant validity of the measurement model.

### 4.2. Structural Model Assessment

**Figure 2:**
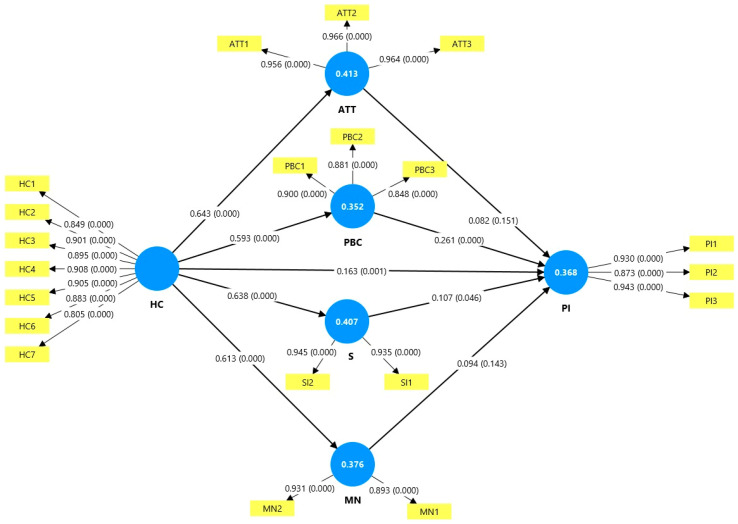
Structural model for results of structural equation modeling with standardized path coefficients and significance levels.

After the discriminant, convergent, and reliability tests were finished, the structural model was evaluated using the PLS bootstrapping procedure with a complete result, a subsample of 5000, and a one-tailed *t*-test with a significance threshold of 0.05%. The results of the structural model with the path coefficient, which ought to fall between −1 and +1 [105], are shown in Figure 2.

According to Chin [121], the R^2^ values of 0.67, 0.33, and 0.19 represent significant, moderate, and weak measures, respectively. In this study, the model explains 36.8% of the variance in the purchase intention (R^2^ = 0.368), indicating a moderate explanatory power. This R^2^ value demonstrates that the variables examined in this research explain an acceptable percentage of healthy food purchase intention variance. The model’s overall fit was assessed using the root mean square residual (SRMR), which yielded a value of 0.048, well below the recommended threshold value of 0.080 [115]. This confirms the measurement model’s adequate fit and supports the validity of the relationships identified in the structural analysis.

The hypothesis test results reveal significant patterns in the relationship between health consciousness and healthy food purchase intentions (Table 6 and Figure 2). Figure 2 illustrates our structural model with standardized path coefficients (β) and their significance levels, with thicker lines representing stronger relationships between constructs. The analysis demonstrates that health consciousness has substantial and statistically significant direct effects on all mediating variables: attitude (β = 0.643, *p* < 0.001), perceived behavioral control (β = 0.593, *p* < 0.001), self-identity (β = 0.638, *p* < 0.001), and moral norms (β = 0.613, *p* < 0.001). These relationships, represented by hypotheses H1a through H1d, exhibit robust t-statistics (ranging from 18.866 to 22.467), indicating health consciousness’ strong and consistent influence on these psychological constructs. However, the subsequent effects of these mediating variables on the purchase intention show varying levels of significance and strength. Perceived behavioral control demonstrates the strongest significant impact on the purchase intention (β = 0.261, *p* < 0.001), followed by self-identity (β = 0.107, *p* = 0.046). Contrary to expectations, the effects of attitude (β = 0.082, *p* = 0.151) and moral norms (β = 0.094, *p* = 0.143) on the purchase intention were not statistically significant, leading to the rejection of hypotheses H2a and H2d. Notably, health consciousness maintains a significant direct effect on the purchase intention (β = 0.163, *p* = 0.001), suggesting that while some mediation exists through perceived behavioral control and self-identity, health consciousness also influences the purchase intention through direct pathways.

## 5. Discussion

This study examines the influence of health consciousness on healthy food purchase intentions through multiple psychological mechanisms. The findings reveal complex patterns in how health consciousness shapes consumer behavior regarding healthy food purchases.

The non-significant effect of attitude on the purchase intention contrasts with traditional TPB research, which typically identifies attitudes as primary determinants of intentions. Several contextual factors may explain this unexpected finding. First, for young, educated consumers in emerging markets like Peru, practical considerations concerning perceived control (accessibility, affordability, and convenience) may outweigh attitudinal factors when translating health consciousness into purchase intentions. Second, the prominent role of self-identity suggests that for this demographic group, purchase decisions are more strongly driven by how these choices align with their self-concept than by general attitudinal evaluations. Additionally, cultural factors in Latin American contexts may emphasize different psychological pathways in consumer decision making compared to the Western contexts where the TPB was primarily developed and tested.

The results support hypotheses H1a through H1e, demonstrating strong direct effects of health consciousness on psychological mediators. Health consciousness shows a robust influence on attitude formation (H1a: β = 0.643, *p* < 0.001), aligning with previous studies that identify health consciousness as a key determinant of positive attitudes towards healthy foods [2,68]. Similarly, the significant effect on perceived behavioral control (H1b: β = 0.593, *p* < 0.001) supports earlier findings that health-conscious individuals exhibit greater perceived control over their food choices [35,51,107].

The strong relationship between health consciousness and self-identity (H1c: β = 0.638, *p* < 0.001) confirms previous research suggesting that health consciousness plays a crucial role in developing health-oriented self-identities [43]. This finding reinforces that individuals’ health consciousness significantly contributes to how they view themselves as health-conscious consumers. Similarly, the significant effect on moral norms (H1d: β = 0.613, *p* < 0.001) aligns with research indicating that health consciousness influences the development of moral frameworks around food choices [47,49]. However, the results reveal a more complex picture when examining the relationships between mediating variables and purchase intention (H2a–H2d). Contrary to expectations and previous research [17,66,90,91], attitude does not significantly influence the purchase intention (H2a: β = 0.082, *p* = 0.151). Similarly, the non-significant effect of moral norms on the purchase intention (H2d: β = 0.094, *p* = 0.143) challenges previous findings about the role of moral considerations in food choices.

In contrast, perceived behavioral control emerges as the strongest predictor of purchase intentions among the mediating variables (H2b: β = 0.261, *p* < 0.001), supporting research that emphasizes the importance of the perceived ability and resources in healthy food choices [17,66,90,91]. Self-identity also shows a significant, though modest, effect on purchase intentions (H2c: β = 0.107, *p* = 0.046), suggesting that consumers’ self-perception as health-conscious individuals contributes to their purchase intentions. The direct effect of health consciousness on purchase intention (β = 0.163, *p* = 0.001) indicates that health-conscious consumers may form purchase intentions through both direct and indirect pathways. This finding aligns with recent studies identifying health consciousness as a fundamental driver of healthy food purchase decisions [2,79].

### 5.1. Theoretical Contributions

This research makes three significant theoretical contributions to the literature on consumer behavior and healthy food consumption. First, it extends the Theory of Planned Behavior by positioning health consciousness as an antecedent variable that influences multiple psychological determinants simultaneously rather than treating it as a moderator or isolated factor. This approach provides a more comprehensive understanding of how fundamental traits like health consciousness shape the entire psychological decision-making framework. Second, our findings challenge traditional TPB assumptions about the primacy of attitudes in behavioral intentions by demonstrating that perceived behavioral control and self-identity have superior explanatory power in the context of healthy food choices. Third, this research provides a more nuanced understanding of the psychological mechanisms through which health consciousness influences purchase decisions in emerging markets, particularly in Latin American contexts that have been under-represented in previous research.

The results have important theoretical implications for the Theory of Planned Behavior in the context of healthy food consumption, suggesting that the relative importance of its components may vary depending on the specific context and behavior being studied. These findings extend current theoretical frameworks by demonstrating that the pathways through which health consciousness influences purchase intentions in emerging markets with young consumers differ from those typically observed in Western contexts.

### 5.2. Managerial Implications

From a managerial perspective, these findings contrast traditional marketing approaches, focusing primarily on attitude change through information provision and persuasion.

For example, previous studies like those by Carfora et al. [39] and Judge et al. [27] emphasized the importance of attitude-focused interventions; our results suggest that marketing strategies should prioritize enhancing consumers’ perceived behavioral control and strengthening their health-oriented self-identity. This could involve providing practical guidance on healthy food selection, preparation methods, and accessibility while helping consumers develop and maintain a health-conscious self-image.

Marketing communications should address both practical barriers to healthy food consumption and the identity-related aspects of health-conscious consumption.

The results suggest that simply creating positive attitudes toward healthy foods may not be sufficient to drive purchase intentions. Instead, brands should empower consumers by enhancing their perceived control over healthy food choices and supporting the development of health-oriented identities. Understanding these complex relationships can benefit public health initiatives and food companies. Educational programs could enhance health consciousness while addressing perceived barriers to healthy food consumption. By recognizing that health consciousness operates through multiple pathways, interventions can be designed to target both direct and indirect influences on purchase intentions.

### 5.3. Limitations and Future Research

The present study has some limitations that should be acknowledged. First, while the measurement model demonstrates robust psychometric properties, the cross-sectional nature of the data prevents drawing causal inferences about the relationships between health consciousness and purchase intentions. Second, our research focuses on purchase intentions rather than actual purchasing behavior, which may not fully capture the complexity of the intention–behavior gap in healthy food consumption. Additionally, methodological limitations include the reliance on self-reported measures, which may be subject to social desirability bias, particularly when assessing health consciousness and moral norms. While the structural model explains a significant portion of the variance in purchase intentions (R^2^ = 0.368), other potentially important variables might not have been captured in our framework. Finally, the non-significant relationship between attitude and purchase intention should be interpreted within this study’s specific demographic and cultural context, rather than as a broader challenge to the established TPB framework. Future research should examine whether similar patterns emerge across different age groups, educational backgrounds, and cultural contexts.

Several promising directions emerge from our findings for future research. Longitudinal studies would be valuable to examine how the relationship between health consciousness and purchase intention evolves, track the development and stability of health-oriented self-identity, and investigate the dynamic nature of perceived behavioral control in healthy food choices. Future research could also benefit from incorporating additional mediating variables, such as nutritional knowledge and food literacy, while exploring the potential moderating effects of demographic variables and situational factors. Examining contextual factors such as food availability and pricing could provide additional insights into the complex nature of healthy food purchase decisions.

Including actual purchase behavior in future studies would be particularly valuable in validating the intention–behavior relationship. This could be accomplished through experimental designs and diary studies to capture real-world food purchasing decisions. Furthermore, comparative research across different cultural contexts and food categories would help examine the generalizability of our findings and identify potential differences between various consumer segments. Understanding how external barriers to healthy food consumption and social support influence the health consciousness–intention relationship would also provide valuable insights for developing more effective interventions.

The role of the food retail environment and its influence on the effectiveness of health consciousness represents another critical area for future investigation. Research in this direction could help bridge the gap between individual psychological factors and environmental influences on healthy food purchasing behavior. Such studies would contribute to a more comprehensive understanding of how health consciousness influences healthy food purchase decisions and help develop more effective interventions to promote healthy eating behaviors.

## 6. Conclusions

This research provides novel insights into the complex relationship between health consciousness and healthy food purchase intentions. The findings reveal that health consciousness operates through multiple psychological pathways, challenging traditional assumptions about consumer decision making in healthy food choices. This study demonstrates that the translation of health consciousness into purchase intentions is more nuanced than previously understood, with some psychological mechanisms proving more influential than others.

This research significantly identifies key psychological mechanisms through which health consciousness influences purchase decisions. The findings highlight the primacy of perceived behavioral control and self-identity in shaping purchase intentions, suggesting a shift from traditional attitude-centric approaches to more comprehensive models of health-conscious consumption. These insights are particularly relevant in the current global context, where increasing health awareness reshapes food consumption patterns. The findings suggest that the successful promotion of healthy food consumption requires strategies that address both practical barriers and identity-related aspects of food choice, offering valuable guidance for food industry stakeholders and public health initiatives to promote healthier eating habits.

## Figures and Tables

**Table 1:** Summary of research hypotheses.

Hypothesis	Key References
H1a: Health consciousness positively impacts attitude toward healthy food.	Health consciousness predicts positive attitudes toward health-oriented products. Research across cultural contexts shows it is one of the most significant factors shaping attitudes toward nutritious eating habits [2,51].
H1b: Health consciousness positively impacts perceived behavioral control regarding healthy eating.	Health-conscious individuals demonstrate greater perceived control over their dietary choices. They maintain their health through nutrition and show increased confidence in identifying and selecting healthy options [10,65,107].
H1c: Health consciousness positively impacts healthy food purchase intention.	Health consciousness is a key factor in buying decisions for health-oriented products. Studies identify it as one of the most significant motivating factors for healthy food consumption, particularly during health-critical periods [2,20,65].
H1d: Health consciousness positively impacts self-identity regarding healthy eating.	Health consciousness shapes how consumers view themselves. Concern about personal/family health invokes interest in seeking information about healthy food, leading to health-oriented self-identities [42,43,108].
H1e: Health consciousness positively impacts moral norms regarding healthy eating.	Consumers develop ethical frameworks around food choices that extend beyond nutritional properties. Health-conscious individuals categorize foods ethically and translate food decisions into moral judgments [47,49].
H2a: Attitude positively impacts healthy food purchase intention.	Attitude is traditionally considered a strong predictor of purchase intention for health-oriented products. Multiple studies demonstrate that positive consumer attitudes significantly influence the likelihood of purchasing healthy foods [17,57,88].
H2b: Perceived behavioral control positively impacts healthy food purchase intention.	Individuals with higher perceived control exhibit stronger intentions to purchase healthy foods. Research shows that clear product understanding and autonomy in food selection enhance purchase intentions [99,100,109].
H2c: Self-identity positively impacts healthy food purchase intention.	Self-identity significantly predicts healthy food purchase intention even when controlling other factors. Identity functions as a self-regulatory mechanism guiding food choices [39,40,41].
H2d: Moral norms positively impact healthy food purchase intention.	Moral considerations significantly influence purchase intentions for healthy and ethically produced foods. Research shows moral norms are crucial determinants of natural food purchase intentions across cultural contexts [44,45,46].

**Table 2:** Sample demographic characteristics.

Variables	Categories	*n*	%
Gender	Male	200	34.9
	Female	373	65.1
Age group	Under 20	261	45.5
	20–24	229	39.9
	25–29	41	7.1
	30–34	22	3.8
	35–39	7	1.2
	40 or above	13	2.5
Education	Graduate studies	60	10.5
	Bachelor’s degree	513	89.5
Marital status	Single	535	93.4
	Married	32	5.6
	Separated	4	0.7
	Partnership	2	0.3
Total		573	100

**Table 3:** Convergent validity results: factor loadings, composite reliability, and average variance extracted for all constructs.

Variable	Code	Loading	Cronbach’s Alpha	Composite Reliability	Average Variance Extracted
Attitude	ATT1	0.956	0.960	0.974	0.926
	ATT2	0.966			
	ATT3	0.964			
Health Consciousness	HC1	0.849	0.950	0.959	0.772
	HC2	0.901			
	HC3	0.895			
	HC4	0.908			
	HC5	0.905			
	HC6	0.883			
	HC7	0.805			
Moral Norms	MN1	0.893	0.799	0.908	0.831
	MN2	0.931			
Perceived Behavioral Control	PBC1	0.900	0.849	0.909	0.768
	PBC2	0.881			
	PBC3	0.848			
Healthy Food Purchase Intention	PI1	0.930	0.904	0.940	0.839
	PI2	0.873			
	PI3	0.943			
Self-identity	SI1	0.935	0.868	0.938	0.883
	SI2	0.945			

**Table 4:** Discriminant validity analysis: heterotrait–monotrait ratio matrix for all study variables.

Variable	Attitude	Health Consciousness	Moral Norms	Perceived Behavioral Control	Healthy Food Purchase Intention	Self-Identity
Attitude						
Health Consciousness	0.672					
Moral Norms	0.806	0.695				
Perceived Behavioral Control	0.753	0.658	0.871			
Healthy Food Purchase Intention	0.527	0.532	0.598	0.628		
Self-identity	0.673	0.702	0.823	0.772	0.560	

**Table 5:** Discriminant validity analysis: Fornell–Larcker for all study variables.

Variable	Attitude	Health Consciousness	Moral Norms	Perceived Behavioral Control	Healthy Food Purchase Intention	Self-Identity
Attitude	0.962					
Health Consciousness	0.643	0.879				
Moral Norms	0.717	0.613	0.912			
Perceived Behavioral Control	0.685	0.593	0.726	0.877		
Healthy Food Purchase Intention	0.498	0.496	0.515	0.553	0.916	
Self-identity	0.616	0.638	0.690	0.664	0.499	0.940

**Table 6:** Structural model results: path coefficients, T-statistics, and *p*-values for hypothesized relationships.

Hypothesis	Path Coefficients (β)	T-Statistics	*p* Values	Decision
H1a: Health consciousness positively impacts attitude toward healthy food.	0.643	22.467	0.000 ***	Accepted
H1b: Health consciousness positively impacts perceived behavioral control regarding healthy eating.	0.593	18.866	0.000 ***	Accepted
H1c: Health consciousness positively impacts self-identity regarding healthy eating.	0.638	22.079	0.000 ***	Accepted
H1d: Health consciousness positively impacts moral norms regarding healthy eating.	0.613	20.771	0.000***	Accepted
H1e: Health consciousness positively impacts healthy food purchase intention.	0.163	3.423	0.001 ***	Accepted
H2a: Attitude positively impacts healthy food purchase intention.	0.082	1.436	0.151	Rejected
H2b: Perceived behavioral control positively impacts healthy food purchase intention.	0.261	4.427	0.000 ***	Accepted
H2c: Self-identity positively impacts healthy food purchase intention.	0.107	1.999	0.046 *	Accepted
H2d: Moral norms positively impact healthy food purchase intention.	0.094	1.465	0.143	Rejected

Note: Statistical significance levels: * *p* < 0.05, *** *p* < 0.001.

## Data Availability

The original contributions presented in the study are included in the article. The data are not publicly available due to privacy concerns related to participant information and ethical restrictions outlined in the research protocol. For further inquiries, please contact the corresponding author.

## Appendix A

**Table A1:** Measurement Items in English and Spanish.

	English Item	Spanish Item
Health Consciousness		
HC1	I reflect a lot about my health.	Reflexiono mucho sobre mi salud.
HC2	I am very conscious about my health.	Soy muy consciente de mi salud.
HC3	I am alert to changes in my health.	Estoy alerta a los cambios en mi salud.
HC4	I take responsibility for the state of my health.	Me hago responsable del estado de mi salud.
HC5	I am aware of the state of my health throughout the day.	Soy consciente del estado de mi salud a lo largo del día.
HC6	I try to choose foods that are good for my health.	Busco elegir alimentos que sean buenos para mi salud.
HC7	I prefer food products without additives.	Prefiero los productos alimenticios sin aditivos.
Attitude		
ATT1	Buying healthy foods is a good idea.	Comprar alimentos saludables es una buena idea.
ATT2	Buying healthy foods is important.	Comprar alimentos saludables es importante.
ATT3	Buying healthy foods is beneficial.	Comprar alimentos saludables es beneficioso.
Perceived Behavioral Control		
PBC1	I have the ability to buy healthy foods.	Tengo la capacidad para comprar alimentos saludables.
PBC2	I have resources to buy healthy foods.	Tengo recursos para comprar alimentos saludables.
PBC3	I have time and willingness to buy healthy foods.	Tengo tiempo y disposición para comprar alimentos saludables.
Moral Norms		
MN1	I feel obligated to buy healthy foods.	Siento la obligación de comprar alimentos saludables.
MN2	I feel that consuming healthy foods makes me feel good.	Siento que consumir alimentos saludables me hace sentir bien.
Self-Identity		
SI1	I am a consumer of healthy foods.	Soy un consumidor de alimentos saludables.
SI2	Consuming healthy foods is an important part of who I am.	Consumir alimentos saludables es una parte importante de mi manera de ser.
Purchase Intention		
PI1	I intend to purchase healthy foods in my next shopping trip.	Tengo la intención de comprar alimentos saludables en mi próxima compra.
PI2	I intend to pay even a little more for healthy foods.	Tengo la intención de pagar incluso un poco más por alimentos saludables.
PI3	Whenever I need to buy food, I look for healthy options.	Siempre que tenga necesidad de comprar alimentos, busco que sean saludables.

Note: All items were measured on a 5-point Likert scale, where 1 = Strongly disagree and 5 = Strongly agree.
